# Long-term Follow-up of Patients Undergoing Nephrectomy for Urolithiasis

**DOI:** 10.1590/S1677-5538.IBJU.2024.0375

**Published:** 2025-01-10

**Authors:** Thiago Augusto Cunha Ferreira, Alexandre Danilovic, Samirah Abreu Gomes, Fabio Carvalho Vicentini, Giovanni Scala Marchini, Fábio César Miranda Torricelli, Carlos Alfredo Batagello, William Carlos Nahas, Eduardo Mazzucchi

**Affiliations:** 1 Universidade de São Paulo Faculdade de Medicina Hospital das Clínicas HCFMUSP SP Brasil Hospital das Clínicas, Faculdade de Medicina, Universidade de São Paulo - HCFMUSP, SP, Brasil; 2 Universidade de São Paulo Faculdade de Medicina Departamento de Clínica Médica São Paulo Brasil Departamento de Clínica Médica, Laboratório de Nefrologia Celular, Genética e Molecular Faculdade de Medicina da Universidade de São Paulo, São Paulo, Brasil

**Keywords:** Urolithiasis, Nephrectomy, Glomerular Filtration Rate

## Abstract

**Purpose:**

This prospective study aimed to identify risk factors associated with progression to stage 3 chronic kidney disease (CKD) and the occurrence of kidney stone formation or growth following nephrectomy for urolithiasis.

**Materials and methods:**

From January 2006 to May 2013, patients undergoing nephrectomy for urolithiasis were enrolled. Renal function was assessed using estimated glomerular filtration rate (eGFR) via the Chronic Kidney Disease Epidemiology Collaboration equation, while kidney stone events were detected using computed tomography.

**Results:**

Among 107 patients followed for an average of 83.5 months, type 2 diabetes mellitus (T2DM) significantly increased the risk of progression to stage 3 CKD by 34.79-fold (p=0.004). Age was associated with a 15% increase in the odds of developing stage 3 CKD per year (p=0.01), while higher preoperative eGFR was protective (OR=0.84, p<0.01). DMSA-99mTc values below 15% were less likely to lead to renal function deterioration. New kidney stone formation occurred in 15.9% of patients and stone growth observed in 12.1%. Contralateral kidney stones (p<0.01) and hypercalciuria (p=0.03) were identified as risk factors for kidney stone events.

**Conclusions:**

T2DM and age were predictors of CKD progression, while higher preoperative eGFR was protective. Hypercalciuria and contralateral kidney stones increased the risk of kidney stone formation and/or growth post-nephrectomy for urolithiasis.

## INTRODUCTION

Urolithiasis is a common and recurring disease that can impair patient's quality of life (1). Several studies have demonstrated an increased risk of chronic kidney disease (CKD) in patients with urinary calculi (2-4). The causes are probably multifactorial and include the cumulative effects of transient renal obstruction during the stone passage, secondary infection, renal injury due to urolithiasis treatments, and the deposition of intratubular crystals causing interstitial inflammation and tubular cell damage (5-7). Despite advances in minimally invasive procedures, nephrectomy is still used for the treatment of kidney stones in case of irreversible loss of renal function associated with pain or severe life-threatening urinary infections (8-10).

It is crucial to predict renal function progression and the likelihood of kidney stone recurrence after nephrectomy to make informed treatment decisions and closely monitor high-risk patients (11). Our hypothesis is that these patients already affected by urolithiasis have specific risk factors for deterioration of renal function and recurrence of kidney stones. We aimed to identify the risk factors for progression to stage 3 CKD and new stone formation or growth of preexisting kidney stones in the contralateral kidneys of patients undergoing nephrectomy for urolithiasis.

## MATERIALS AND METHODS

We prospectively observed a cohort that included patients who underwent nephrectomy for urolithiasis between January 2006 and May 2013. The exclusion criteria were: [1] follow-up period <6 months, [2] recurrent tract infections after nephrectomy, [3] spinal cord injuries that compromised mobility, or [4] refused participation in the study. Total nephrectomy for urolithiasis was indicated for patients who experienced the loss of renal function accompanied by pain or recurrent urinary tract infection as well as for those who presented with severe infections associated with urinary focus sepsis (12, 13). After being instructed by the researchers about all stages of this study, all patients signed a Free and Informed Consent Form. This study was approved by the Ethics Committee (no. 63637416.6.0000.0068) and was conducted in accordance with the ethical principles and the Helsinki Declaration guidelines.

### Preoperative assessment

We evaluated the comorbidities of the patients using the age-adjusted Charlson Comorbidity Index, and the surgical risk was stratified according to the American Society of Anesthesiologists (ASA) classification. In order to assess stone status and the relative function of each kidney, all patients underwent preoperative abdominal and pelvic computed tomography (CT) and static renal scintigraphy with dimercaptosuccinic acid (DMSA-99mTc).

### Follow-up

The patients were followed up to November 2019 through periodic medical appointments, occurring quarterly in the first 6 months after surgery and annually for the remainder of the follow-up period. The estimated glomerular filtration rate (eGFR) was calculated using the Chronic Kidney Disease Epidemiology Collaboration (CKD-EPI) equation. At least one valid 24-hour (h) urine sample was obtained 6 months after nephrectomy to measure calcium, oxalate, citrate, and uric acid levels in all patients. A sample was considered valid in the absence of urinary tract infection, with no diuretics, potassium citrate, or allopurinol use, no hormone replacement, and the presence of adequate values of creatinine (1,040-2,350 mg/24 h for men and 740-1,570 mg/24 h for women). The abnormal values of the 24-h urinary parameters according to the reference values of the enzymatic assays used in our institution were as follows: hypercalciuria, >300 mg/24 h of calcium excretion for men and >250 mg/24 h for women; hypocitraturia, <320 mg/24 h citrate excretion; hypernatriuria, >220 mEq/24 h sodium excretion; hyperoxaluria, >31 mg/24 h of oxalate excretion; and hyperuricosuria >800 mg/24 h of uric acid excretion for men and > 750 mg/24 h for women.

Metabolic abnormalities were managed according to the institutional protocol during follow-up, with hydrochlorothiazide titrated up to 50 mg/day for patients with idiopathic hypercalciuria and potassium citrate at variable doses from 20-60 mEq/day for patients with hypocitraturia (14). Chemical analyses were performed on the urinary stones to determine their composition.

Kidney and urinary tract ultrasonography was performed once a year for all patients. In cases where the growth or formation of a new urinary stone was observed, a non-contrast-enhanced CT scan was performed for confirmation (15-17). A new event related to urolithiasis in the postoperative follow-up was defined as the formation or growth of renal calculi in the remaining kidney, as confirmed by CT (18).

### Statistical analysis

Statistical analysis was conducted using R statistical software (19). In the descriptive analysis of demographic data, numeric variables are reported as mean values and standard deviations, while categorical variables are presented as absolute numbers and percentages. To identify predictors of kidney function and new urolithiasis events after nephrectomy, a logistic regression model was developed using the purposeful selection method proposed by Hosmer and Lemeshow for selecting variables (20). McFadden's pseudo coefficient of determination (pseudo-R^2^) was used to assess the accuracy of the model (21). The significance level used for the final model was 5%, and a 25% significance level was used for the preliminary univariate analysis. We employed the Kaplan−Meier estimator to analyze censored curves by progression to CKD stage 3 and new urolithiasis events, and the log-rank test was used to compare related variable curves (22, 23). To predict the final GFR and CKD-EPI values, we developed a mathematical model using multivariate linear regression, employing the backward variable selection method and calculating the Akaike Information Criterion (AIC) value (20). The quality of the model was ensured through residual analysis and the determination coefficient R^2^.

## RESULTS

During the study period, 150 patients underwent nephrectomy for urolithiasis. We excluded three patients with spinal cord injuries. After nephrectomy, 40 patients did not meet the necessary follow-up criteria due to discontinuation of outpatient follow-up before 6 months or failure to return for the requested complementary tests. Therefore, these patients were excluded from the study.

### Preoperative demographic and clinical characteristics

Of 107 patients, 78.5% (84/107) were women. The average BMI was 26.9 kg/m2. Type 2 diabetes mellitus (T2DM) and systemic arterial hypertension (SAH) were detected in 15% and 50.9% of patients, respectively. Furthermore, 51.4% and 36.4% of patients were classified with ASA 2 score and Charlson comorbidity index ≥ 1, respectively. Forty-nine patients (45.8%) patients had stage 2 CKD, with an overall mean eGFR of 73.48 (±29.41) mL/min/1.73m². The DMSA-99Tc value of the affected kidneys was < 15% in 83 patients. Table-1 provides additional information on recruited patients.

**Table 1:** Preoperative and follow-up data after nephrectomy for urolithiasis.

Variable		Value
Patients, N		107
Female, N (%)		84 (78.5%)
Age, years (mean ± SD)		49 (±13)
Black race, N (%)		54 (50.5%)
BMI (Kg/m^2^), (mean ± SD)		26.9 (±5.2)
SAH, N (%)		54 (50.9%)
Diabetes Mellitus, N (%)		16 (15%)
Affected kidney side, left, N (%)		53 (49.5%)
Preoperative infecction, N (%)		70 (65.4%)
Charlson ≥1, N (%)		39 (36.4%)
ASA, N (%)		
	1	27 (25.2%)
	2	55 (51.4%)
	3	22 (20.6%)
	4	3 (2.8%)
Affected kidney DMSA-99Tc, (mean ± SD)		8.8 (±9.7)
Affected kidney DMSA-99Tc < 15%, N (%)		83 (77.6%)
CKD stage N (%)		
	1	31 (29%)
	2	49 (45.8%)
	3	16 (15%)
	4	2 (1.9%)
	5	9 (8.4%)
eGFR (mL/min/1.73m²), (mean ± SD)		73.4 (±29.4)
Contralateral kidney stone, N (%)		35 (32.7%)
Follow up time, months (mean ± SD)		83.5 (±35.5)
Final eGFR (mL/min/1.73m²), (mean ± SD)		67.3 (±27.4)
Outcome		
	Stage improvement, N (%)	15 (14%)
	Stage worsens, N (%)	32 (29.9%)
	Stage maintenance, N (%)	60 (56.1%)
Albuminuria >30 mg/g, N (%)		53 (49.5%)
Dialysis, N (%)		6 (5.6%)
New urolithiasis event, N (%)		30 (28%)
Urinary metabolic disorder, N (%)		78 (72.9%)
Hypocitraturia, N (%)		71 (66.4%)
Hypercalciuria, N (%)		17 (15.9%)
Hyperoxaluria, N (%)		5 (4.7%)
Hypernatriuria, N (%)		6 (5.6%)
Hyperuricosuria, N (%)		6 (5.6%)
Cystinuria, N (%)		2 (1.9%)
Urinary pH, (mean ± SD)		6.5 (±0.7)
Urinary volume, mL/24h (mean ± SD)		1,874.3 (±529.3)

N, number; ST, standard deviation; BMI, body mass index; SAH, systemic arterial hypertension; ASA, *American Society of Anesthesiologist*; DMSA-99mTc, dimercaptosuccinic acid; CKD, chronic kidney; eGFR, estimated glomerular filtration rate;

### Nephrectomy

Nephrectomy was performed laparoscopically in 62 patients (57.9 %). Staghorn stones accounted for 67.3% of the kidney stones removed, while ureteral and pelvic stones were responsible for nephrectomy in 19 (17.8%) cases. An anatomopathological report of the removed kidneys revealed chronic pyelonephritis, xanthogranulomatous pyelonephritis, pyonephrosis, and renal atrophy in 36 (33.6%), 26 (24.3%), 22 (20.6%), and 15 (14.0%) patients, respectively. Stone composition analysis showed that 67 (62.6%) of the removed stones were composed of calcium, of which 83.5% were exclusively composed of calcium oxalate and 16.5% were composed of calcium phosphate. Struvite was observed in 56 (52.3%) cases (alone or in combination), while two (1.9%) cystine stones were evident.

### Follow-up

The average follow-up period was 83.5 (±35.5) months. During this period, 32 (29.9%) patients experienced a decline in the CKD stage, 53 (49.5%) had albuminuria levels exceeding 30 mg/g, and six (5.6%) progressed to dialysis. Additionally, 30 patients (28.0%) reported new occurrences of urolithiasis. Of the total patients, 78 (72.9%) had at least one urinary metabolic alteration that increased the risk of urolithiasis. Hypocitraturia was the most common alteration, accounting for 66.4% of the cases (Table-1).

### Analysis of predictors for progression to stage 3 CKD

Univariate analysis was conducted using preoperative variables of patients with stage 1 or 2 CKD before nephrectomy.

Following the initial univariate analysis, only the variables that yielded a likelihood test p-value of < 0.25 were considered for the model. A purposeful selection of variables was conducted, leading to the final model, as presented in Table-2. T2DM was identified as a significant risk factor, resulting in a 34.79-fold increase in the likelihood of progression to stage 3 CKD. Furthermore, for every additional preoperative eGFR unit, the risk of progression to stage 3 CKD decreased by 0.84 folds. Finally, the odds ratio for developing stage 3 CKD increased by 15% every year.

**Table 2:** Univariate and multivariate analysis of stage 3 CKD progression predictors - preoperative variables.

Univariate			Multivariate				
Variable	Progression to stage 3 of CKD. n 80		p1				
	Yes (N = 12)	No (N = 68)		Coefficient	Odds ratio (OD)	95%CI	P value
Female	8 (66.6%)	56 (82.3%)	0.2	-1,9	0.14	0.01 – 1.18	0.09
Age, years (mean ± SD)	59.5 (±13.6)	45.4 (±10.9)	0.004	0.14	1.15	1.04 – 1.31	0.01
BMI (Kg/m2), (mean ± SD)	27.9 (±6.9)	26.8 (±4.7)	0.6				
Black race, N (%)	6 (50%)	33 (48.5%)	0.9				
Diabetes Mellitus, N (%)	6 (50%)	6 (8.8%)	0.001	3.55	34.79	4.15 – 606.26	0.004
SAH, N (%)	8 (66.6%)	28 (41.8%)	0.1				
ASA 1, N (%)	1 (8.3%)	25 (36.7%)	0.06				
ASA 2, N (%)	8 (66.6%)	37 (54.4%)					
ASA >3, N (%)	3 (25%)	6 (8.8%)					
Charlson >1, N (%)	6 (50.00%)	11 (16.1%)	0.01				
Preoperative infection, N (%)	6 (50%)	46 (67.6%)	0.2				
eGFR (mL/min/1.73m²) (mean ± SD)	68.4 (±10.5)	88.8 (±19.7)	<0.001	-0.17	0.84	0.72 – 0.94	0.007
Stage 1 of CKD, N (%)	1 (8.3%)	30 (44.1%)	0.01				
Stage 2 of CKD, N (%)	11 (91.6%)	38 (55.8%)					
Affected kidney DMSA-99Tc (mean ± SD)	11.75 (±9.3)	7.7 (±7.4)	0.1				
Contralateral kidney stone, N (%)	4 (33.3%)	18 (26.4%)	0.6				

^1^ Likelihood ratio test for categorical variables and t-test for numerical variables. Hosmer-lemeshow test, p=0.575; pseudo-R² = 0.57. SD = Standard deviation; BMI = body mass index; N = number; SAH = systemic arterial hypertension; ASA = *American Society of Anesthesiologist;* CKD = chronic kidney disease; eGFR = estimated glomerular filtration rate; DMSA-99mTc = dimercaptosuccinic acid. 95%CI, 95% confidence interval.

### DMSA-99mTc cut-off point

**Figure 1:**
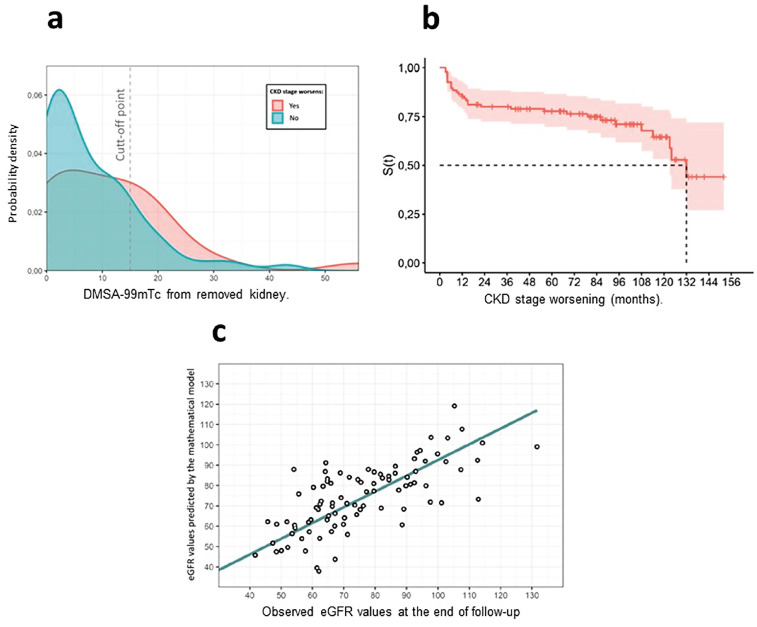
a) Probability density of worsening kidney function as a function of the DMSA value of the removed kidney. Kaplan Meier curve for CKD stage worsening. DMSA-99mTc values lower than 15% being less likely to cause long-term worsening of renal function; b) Kaplan Meier curve for CKD stage worsening. The shaded area in salmon represents the 95% confidence interval; c) Distribution of observed values by predicted estimated glomerular filtration rate (eGFR) values at the end of follow-up – mean time. The blue line represents the identity function.

We proposed a DMSA-99mTc cut-off point to aid in decision-making regarding patient management and to assess the impact of removal on long-term renal function. We performed logistic regression of the preoperative variables using a worsening CKD stage as an outcome. To evaluate the linearity of the numerical variable DMSA-99mTc, we transformed it into a categorical variable and established a cutoff point by separating it into quartiles. The suggested cutoff point was 15%, owing to the lower p-value. This value was found to illustrate the most significant difference in behavior through the probability density function, with DMSA-99mTc values lower than 15% being less likely to cause long-term worsening of renal function – Figure-1a.

### Kaplan Meier curve for CKD stage worsening

Figure-1b depicts the Kaplan−Meier curve estimate of the time taken in months for the CKD stage to worsen. The median curve function was 132 months, indicating a 50% probability of a patient experiencing a worsening stage of CKD after 132 months.

### Mathematical model for the eGFR value at the end of follow-up.

Table-3 displays the results of both univariate and multivariate analyses of the final model. In this regression, the p-value from the univariate analysis may not necessarily determine the significant variables because of the influence of multicollinearity with other variables in the multivariate model and the presence of confounding effects. The final model obtained is given by the following equation:


$$\begin{array}{l} \text{Final eGFR}=\;+8.7+0.77^{*}\text{preoperative}\\ \text{eGFR+0}.\text{33*BMI-1}.\text{2*SAH-10}.\text{61*Charlson}>\text{1-0}.\text{2*DMSA} \end{array}$$

**Table 3:** Multivariate linear regression analysis for final eGFR value - preoperative variables.

Univariate				Multivariate		
Variable	Coefficient	95%CI	p	Coefficient	95%CI	p
Female	6.5	-6.2; 19.41	0.3			
Age	-0.6	-1.06; −0.29	<0.001			
Black race	4.3	-6.20; 14.91	0.4			
BMI (Kg/m^2^)	-0.04	-1.05; 0.98	0.9	0.3	-0.18; 0.84	0.2
Diabetes Mellitus	-8.7	-23.47; 6.04	0.2			
SAH	-14.8	-25.17; −4.59	0.005	-1.2	-6.74; 4.33	0.6
ASA, 2	-13.3	-24.03; −2.74	0.01			
ASA, 3 e 4	-43.3	-55.90; −30.75	<0.001			
Charlson>1	-32.8	-41.86; −23.91	<0.001	-10.6	-16.91; −4.31	0.001
Preoperative infection	1.3	-9.74; 12.52	0.8			
eGFR (mL/min/1.73m²)	0.7	0.68; 0.88	<0.001	0.7	0.65; 0.88	<0.001
Affected kidney DMSA-99mTc	-0.2	-0.81; 0.28	0.3	-0.2	-0.47; 0.07	0.1
Contralateral Kidney stone	-7.12	-18.33; 4.08	0.2			

R² = 0.77.95%CI, 95% confidence interval; BMI, body mass index; ASA*, American Society of Anesthesiologist*;; SAH, systemic arterial hypertension; CKD, Chronic kidney disease; eGFR, estimated glomerular filtration rate; DMSA-99mTc, dimercaptosuccinic acid.

Figure-1c shows the scatter plot comparing the predicted and observed eGFR values. Pearson's correlation coefficient obtained was 0.78 with p ≤ 0.001.

### New urolithiasis events

We developed a logistic regression model using the purposeful selection of variables method to determine the final model of risk factors for the occurrence of new urolithiasis-related events during patient follow-up. Among the patients, 30 had a new event, with 13 (43.3%) having preexisting calculus enlargement and 17 (56.7%) forming new calculi.

Table-4 presents the univariate analysis and final model for the occurrence of a new urolithiasis-related event during patient follow-up. The variables included the presence of contralateral kidney stones and hypercalciuria. The presence of a contralateral stone increased the chance of a patient experiencing a new event by 3.24 folds.

**Table 4:** Univariate and multivariate analysis of risk factors for the occurrence of new urolithiasis event.

Univariate				Multivariate^2^			
				Coefficient	Odds ratio	95%CI	p
Variable	Ocurrence of new urolithiasis event	p^1^					
	Yes (N = 30)	No (N = 77)					
Female, N (%)	22 (73.3%)	62 (80.5%)	0.4				
Age (mean ± SD)	47.1 (±14.5)	49.7 (±12.4)	0.4				
BMI (Kg/m^2^), (mean ± SD)	26.5 (±6)	27 (±4.9)	0.6				
Black race, N (%)	17 (56.6%)	37 (48%)	0.4				
Diabetes Mellitus, N (%)	4 (13.3%)	12 (15.5%)	0.7				
Preoperative infection, N (%)	20 (66.6%)	50 (64.9%)	0.8				
eGFR (mL/min/1.73m²), (mean ± SD)	71.1 (±31.2)	74.3 (±28.8)	0.6				
Transfusion, N (%)	4 (13.3%)	5 (6.4%)	0.2				
Preoperative contralateral Kidney stone, N (%)	16 (53.3%)	19 (24.6%)	0.005	1.17	3.24	1.31 – 8.15	0.01
Unrinary metabolic disorder, N (%)	25 (83.3%)	52 (67.5%)	0.09				
Hypercalcuria, N (%)	9 (30%)	8 (10.3%)	0.01	1.19	3.3	1.08 – 10.28	0.03
Hypocitraturia, N (%)	23 (76.6%)	48 (62.3%)	0.1				
Hyperuricosuria, N (%)	1 (3.3%)	5 (6.4%)	0.5				
Urinary pH (mean ± SD)	6.6 (±0.7)	6.4 (±0.8)	0.6				
Urinary volume (mL), (mean ± SD)	1,854.3 (±482.1)	1,882.1(±549.3)	0.7				

^1^ Likelihood ratio test for categorical variables and T test for numerical variables.^2^ Hosmer-Lemeshow test, p=0.530; pseudo-R² = 0.10. N, number; %; SD, standard deviation; BMI, body mass index; eGFR, estimated glomerular filtration rate; DMSA-99mTc, dimercaptosuccinic acid.95%CI, 95% confidence interval.

## DISCUSSION

Currently, this is the first prospective study in literature with a long-term follow-up of patients who underwent nephrectomy for urolithiasis. We evaluated 107 adult patients who underwent nephrectomy for urolithiasis in a prospective observational cohort with a mean follow-up period of > 83 months. We found that T2DM and advanced age increased the risk of developing stage 3 CKD, whereas higher initial eGFR provided a protective effect. We also showed that patients with contralateral kidney stones and hypercalciuria had an increased risk of developing new urolithiasis events after nephrectomy. In addition, we developed a mathematical model to predict long-term eGFR in patients who underwent nephrectomy for urolithiasis.

Although urinary lithiasis is more prevalent in males, when evaluating cases of more complex urinary stones that require hospitalization and surgical interventions, we observe a higher prevalence among female patients (24). When assessing the formation of staghorn calculi, loss of renal function, and nephrectomy due to urolithiasis, women exhibit twice the prevalence compared to men (8). A study evaluating 101 nephrectomies for urolithiasis showed that women more frequently progress to renal function loss due to lithiasis than men (10). The indications for nephrectomy occur more often in patients with complex stones associated with recurrent urinary tract infections, which may have contributed to the predominance of female patients in our study.

Previous research by Lee et al. retrospectively analyzed the recurrence of urinary calculi and renal function in 50 patients who underwent nephrectomy for urolithiasis; however, they excluded patients with contralateral kidney stones and only had a mean follow-up of 70 months (25). Our study found that 32.7% of the patients who underwent nephrectomy also had bilateral stones. Therefore, Lee et al. excluded a significant portion of the patient population analyzed in our study.

Renal function can be directly influenced by several clinical factors, with SAH and T2DM being the main causes of CKD worldwide (26). Recognition of CKD in its early stages and early medical follow-up are fundamental for controlling the evolution of the disease and reducing the need for renal replacement therapy (27-29). Ellis et al. retrospectively evaluated a cohort of 709 nephrectomies for renal cell carcinoma and reported a 59% incidence of CKD up to 12 months after surgery, in addition to an increased risk of CKD in patients of advanced age (odds ratio 1.5 [95% confidence interval 1.4−1.6]) (30). In our cohort, we observed that the presence of T2DM before nephrectomy increased the chances of patients progressing to stage 3 CKD by 34.7 times. Advanced age is another predictor of CKD progression. Assessment of these risk factors led to the creation of a mathematical equation to predict long-term eGFR after nephrectomy with great potential for use in clinical practice, allowing the selection of patients at a greater risk of loss of renal function.

Relative renal uptake on DMSA-99mTc scintigraphy has an excellent correlation with eGFR (31, 32) and has been used in clinical practice to assess the viability of the renal unit, which often supports the decision to perform nephrectomy when associated with a severe infectious process or recurrent pain. There is no consensus on an exact DMSA-99mTc cutoff point that could predict the impact of removing the renal unit on eGFR in the long term in patients with urolithiasis. In our study, we defined a cutoff point for DMSA-99mTc values by adapting the variables to assess the worsening stage of CKD. Evaluating the point where the behavior change presented the greatest statistical difference, a value of 15% was reached, and patients whose kidney was removed had DMSA-99mTc values > 15% and had a greater chance of progressing to a worsening stage of CKD.

Metabolic acidosis resulting from nephron loss increases the risk of kidney stone formation due to its effects on calcium metabolism, leading to hypercalciuria, parathyroid hormone dysregulation, and increased levels of 1,25-(OH)_2_ vitamin D (33). This highlights the importance of identifying risk factors for urolithiasis in patients undergoing nephrectomy. Bagrodia et al. compared radical nephrectomies with nephron-sparing surgeries for renal tumors, demonstrating a significant association between a history of urolithiasis and the development of postoperative kidney stones (34). In our study, the presence of a contralateral stone and hypercalciuria increased the risk of the new urinary stone-related event 3.2 times and 3.3 times, respectively. Lee et al. reported a higher frequency of urinary calculi recurrence in patients with metabolic calculi than in those with infectious (25). These data are demonstrated in our study, with a significant influence of hypercalciuria on the risk of a new event related to urinary calculi.

A predictive model of postoperative renal function can influence the planning and decision to perform a nephrectomy (35-37). To date, our study is the first to propose a predictive model of renal function after nephrectomy for urolithiasis. Multivariate linear regression analysis showed that the variables included in the model were preoperative eGFR, BMI, SAH, Charlson score >1, and the DMSA score of the affected kidney.

This study had several limitations. There were significant losses of patients in the postoperative period due to the long follow-up period, and the sample was heterogeneous, with a greater representation of women, perhaps explained by the trend towards a higher frequency of complex cases of urolithiasis in this population.

## CONCLUSIONS

The findings of our study underscore the importance of early detection, management, and intervention to mitigate the risk of CKD progression, particularly in high-risk populations. Additionally, provides valuable insights for clinicians to identify high-risk patients and plan careful follow-ups and treatments to prevent further kidney damage and urolithiasis recurrence.

## Data Availability

The data sets analyzed during the current study are not publicly available due to non-authorization of the institution where the study was carried out but are available from the corresponding author on reasonable request.
